# Online group music therapy: proactive management of undergraduate students’ stress and anxiety

**DOI:** 10.3389/fpsyt.2023.1183311

**Published:** 2023-04-21

**Authors:** Rachael Finnerty, Sean McWeeny, Laurel Trainor

**Affiliations:** ^1^Department of Psychology, Neuroscience and Behaviour, McMaster University, Hamilton, ON, Canada; ^2^McMaster Institute for Music and the Mind, Faculty of Science, McMaster University, Hamilton, ON, Canada; ^3^Rotman Research Institute, Baycrest Hospital, Toronto, ON, Canada

**Keywords:** mental health, music therapy, stress, anxiety, proactive therapy, cortisol, university students, online group therapy

## Abstract

**Introduction:**

In alignment with the World Health Organization’s (WHO) goal to provide comprehensive and integrated mental health services in community-based settings, this randomized control trial explored the efficacy of online group music therapy as a proactive intervention for reducing stress and anxiety in university students who do not necessarily have a diagnosis.

**Methods:**

The study took place during COVID-19 restrictions. Students who volunteered were randomly assigned to 6 weeks of weekly (1) online active group music therapy, (2) online receptive group music therapy, (3) online group verbal therapy (standard of care), or (4) no-intervention (control group). Students rated their stress (Likert scale) and anxiety [State-Trait Anxiety Inventory, State version (STAI-S)], and provided heart rate variability (HRV) using a phone app, pre and post each therapy session.

**Results:**

STAI-S and Likert stress scores significantly reduced from pre to post 45-min online music therapy sessions, with moderate evidence that these changes did not differ from the standard of care (verbal therapy). HRV results were not analyzed statistically as HRV collection was likely compromised due to challenges of remote collection. Students completed the Perceived Stress Scale (PSS) and provided a hair sample for cortisol analysis before and after the 6-week intervention. Changes in stress from week 1 to week 6 were not observed in the PSS measure; however, cortisol increased significantly in the control group as the term progressed, while it remained relatively stable in the therapy groups, suggesting therapy may lead to greater control of stress. Of participants’ demographic characteristics, music sophistication, personality, and changes in quality of life, only the personality trait of conscientiousness correlated significantly with PSS, suggesting online group therapy may be beneficial for a wide range of university students.

**Discussion:**

The results suggest group music therapy can be as effective as group verbal therapy. Further, the study indicates that online delivery can be achieved effectively, supporting the idea that remote therapy may be a viable option for other populations. While the study should be replicated with a larger multi-site sample, it provides one example toward achieving a health-promoting culture on university campuses, consistent with the mental health goals of the Okanagan Charter.

## 1. Introduction

Despite research demonstrating the role of high stress in adverse health outcomes, including decreased mental and physical health (1–4), and despite anxiety remaining the main concern among students in counseling (5), proactive stress and anxiety reduction are not at the forefront of health care interventions. Research in relation to stress and anxiety predominantly focuses on interventions for individuals in crisis, as opposed to proactive measures to prevent acute and chronic experiences of stress and anxiety. Preventative strategies for undergraduate students are paramount considering that the highest levels of anxiety (33.5%) and depression (27.7%) are observed among younger Canadians (15–39 years of age) in comparison to other age groups (6), with suicide ranking the second leading cause of death among young Canadians (7), and the fourth leading cause of death among youth (15–29 years of age) globally (8). A longitudinal study of over 10,000 students from 15 different universities across Canada reported high student stress over the course of the 2020–2021 academic year which aligned with the COVID-19 pandemic (9). Student support services switched to online platforms as a result of the COVID-19 restrictions, which has led to a more permanent shift in thinking about methods of health care provision; the demand for online interventions is expected to continue (10). The present research explored the efficacy of online group music therapy in comparison to the standard of care (online verbal group therapy) and to a no-intervention control group. We predicted that online group music therapy would provide students with a positively stigmatized alternative for support, as engaging in music was reported as a common activity for self-support by university students during the COVID-19 pandemic (11).

Recognizing that elevated stress among university students has been associated with anxiety and depression (12, 13) shifting the focus to stress prevention could lead to a reduction in anxiety and depression. A meta-analysis reviewing proactive measures of cognitive, behavioral, and mindfulness-based interventions supports this idea (14). Arts-based interventions were explored in this meta-analysis, but there were not enough data on these interventions to be included in the analysis. Without meaningful data, arts-based therapies (e.g., music therapy) cannot be proposed with confidence, further highlighting the need for research in this area. An advantage of music therapy is that it is likely to be less negatively stigmatized because engaging in music is typically considered to be a healthy activity and is not associated with being mentally ill (15–17). This is important as more than 75% of students experiencing significant psychological distress do not seek support as a result of negative stigma (18).

Music therapy initially developed as a health care profession in the 1950s in the USA in response to soldiers experiencing post-traumatic stress disorder (19). The Canadian Association of Music Therapy was established in 1974 as the national professional body that sets the standards and qualifications for music therapists in Canada. Music therapists use music purposely within a therapeutic relationship to support health care goals for all age groups and diagnoses including dementia care, neonatal intensive care, autism, mental illness, and perioperative care. Systematic reviews report positive findings as a result of engaging in music therapy, such as improved verbal fluency, reduced anxiety, reduced depression, reduced pain perception, improved psychosocial measures, and improved motivation for treatment, while also recognizing the need for clinical trials with larger sample sizes, appropriate experimental methodology, and objective measurements of treatment effectiveness in order to substantiate these claims (20–25).

Despite music therapists working with diverse age groups and diagnoses, only three studies have been published to date on the effects of music therapy with university or college students (26–28). Each of these studies reported on the benefits of engaging in music therapy to treat a clinical symptom or developmental difficulty; however, only one of the studies implemented a randomized controlled trial (RCT) design (27) and none included a physiological measure. To our knowledge, no music therapy studies have been conducted with a non-clinical population such as university students, who are likely to experience stress and anxiety. The present study was directed at all undergraduate students, as opposed to only those in crisis or with a diagnosis, thereby investigating the effects of proactive rather than reactive support for stress and anxiety.

Proactive or preventive interventions may be helpful in curbing the increasing numbers of students experiencing a crisis. A meta-analysis of clinical control trials and random control trials exploring the effects of music therapy on both physiological and psychological stress-related outcomes observed a medium to large effect of music therapy on stress related outcomes (29), and highlighted a larger effect for group compared to individual music therapy. Prior to the COVID-19 pandemic, research reporting on the online delivery of music therapy was limited to military veterans (30–32) and adolescents (33, 34). Although the COVID-19 pandemic forced music therapists to shift to online platforms (35), research on the efficacy of this delivery mode since the start of the COVID-19 pandemic remains limited to children and adolescents with visual impairments (36), dementia care (37), and student refugees (27).

In the present online music therapy study, a community music therapy approach was used that takes into account the larger cultural, institutional, and social context of the participants (38). Specifically, this approach aims to reframe participants’ preconceived notions about engaging in mental health supports within campus culture. Recruitment posters advocated for proactive wellness and engaging in online group therapy; and students participating in the therapy groups were presented with the opportunity to recognize that it is normal to experience stress or anxiety, and that it is *ok* to engage in support. The community music therapy approach does not require an intake form, an assessment, or a treatment plan.

The present RCT reports on the first application of online music therapy for proactive wellness with undergraduate university students. The study took place during COVID-19 lockdowns when university classes had transitioned to online. It aimed to explore the efficacy of online group music therapy as a proactive intervention for undergraduate students’ stress and anxiety in comparison to the standard of care (online verbal group therapy) and no intervention. More specifically, this research asked two main questions.

Question 1: *Does participating in a 45-min online group music therapy session reduce stress and anxiety from pre- to post-session in comparison to the corresponding standard of care (online verbal therapy)?*

We hypothesized that significant reductions in stress and anxiety measures would be observed pre- to post-sessions for all therapy groups and that the reductions would not differ significantly between therapy groups. To test our hypotheses, we asked participants in both music therapy groups and the verbal therapy group to complete the State-Trait-Anxiety Inventory, State version (STAI-S), to rate their stress on a five-point Likert scale (Likert Stress), and to record their heart rate variability (HRV) from an app on their phone before and after each online group therapy session. Collecting measures before and after each therapy session provides information about the immediate effects of the intervention on stress and anxiety. This is important as experiencing low levels of stress, even for a short period of time, can have benefits (39). HRV was collected as a physiological measure of autonomic nervous system (ANS) function (40). Greater HRV is associated with increased ability to rapidly cope with uncertain and changing environments (41). However, a comprehensive review of the effects of psychotherapeutic interventions on the hypothalamic pituitary adrenal axis (HPA) and ANS regulation in adult samples with mental disorders reported inconclusive results (42). Here we examined whether HRV was sensitive to potential effects of online group music therapy.

Question 2: *Does participating in 6 weeks of weekly online group music therapy sessions reduce stress in comparison to the corresponding standard of care (online verbal therapy) and a no-intervention control group?*

We hypothesized that reductions in stress measures would be observed from week 1 to week 6 for all three therapy groups with no difference in outcomes between the music therapy groups and the standard of care, and that music therapy would result in a reduction in stress in comparison to the control group. To test our hypotheses, we asked participants in both music therapy groups, the verbal therapy group, and the control group to complete the Perceived Stress Scale (PSS), and to mail in a hair sample for cortisol analysis, in both week 1 and week 6. A previous systematic review of RCTs exploring the effects of music interventions on cortisol revealed that only one music therapy study has measured cortisol before and after an intervention, and it was done via saliva (43). However, the results specific to the music therapy group were not reported due to the small sample size (44). Because cortisol from hair samples reflects total HPA activity in the preceding months and is more stable than saliva or blood samples that are affected by circadian rhythms and day-to-day fluctuations (45), in the present study, hair samples were collected in weeks 1 and 6 to provide retrospective information about participant HPA activity. Collecting cortisol and the PSS measure in weeks 1 and 6 provided information about the longer-term effects of engaging in 6 weeks of weekly online group therapy interventions.

In addition to the main outcome measures, we examined several variables that might potentially moderate the results. In addition to demographic data, these variables included personality traits, musical sophistication, and changes in quality of life over the 6-week period of the study. It is important to examine personality traits, as a meta-analysis showed that personality traits can moderate therapy outcomes (46). Regarding musical sophistication, while there is ample evidence that music can positively affect mental health (47), it is unclear if music sophistication moderates the degree of benefit, or a person’s response to music therapy in comparison to verbal therapy. Changes in quality of life cannot be controlled in a naturalistic setting, but it is important to try to account for any adverse or positive experiences of a physical, psychological, social, or environmental nature that might impact the effects of the therapy. This was particularly important for the present study as many students experienced turmoil as pandemic restrictions were continually changing.

As the objectives of the therapy groups were to proactively manage stress and anxiety, we predicted that a significant reduction in stress and anxiety would be observed across outcome measures in all of the online therapy groups pre- and post- each online therapy session (measured by STAI-S, Likert stress, and HRV), as well as an overall reduction in stress between week 1 and week 6 (measured by PSS and cortisol). We also expected that online group music therapy would be as effective as the standard of care (online verbal therapy) and that all therapies would be more effective than the no-intervention control.

## 2. Materials and methods

### 2.1. Overall study design

A randomized control trial, pretest–posttest study design with four groups was approved by the Hamilton Integrated Research Ethics Board (project #11376). The groups were: (1) online active music therapy group, (2) online receptive music therapy group, (3) online verbal based therapy group (standard of care), and (4) no-intervention control. The study included five blocks and all blocks were completed between September 2020 and February 2022. Each block included all four groups. Within each block, each of the three therapy groups participated in a 45-min therapy session every week for 6 weeks (with the exception of Block 1, which ran for 5 weeks due to a conflict with the exam schedule). Measures of stress and anxiety were taken pre and post each therapy session for each individual in each of the three therapy groups. Measures of stress and cortisol hair samples were taken pre and post the 6 weeks of the study protocol for each individual in all four groups in all five blocks (see details below).

### 2.2. Participants

Participants were full-time undergraduate students, aged 18–24 (*M* = 20 years old), at a Canadian university who agreed to adhere to the therapy group guidelines (Supplementary material 1). The study was originally designed with five different groups: (1) online active music therapy group, (2) online receptive music therapy group, (3) online verbal therapy group, (4) wait-listed group, and (5) no-intervention control group. Due to challenges recruiting participants during the COVID-19 pandemic, the waitlist group was removed from the study. Groups were to be run with weekly sessions for 6 weeks, with 10 participants per group. Thus, a block of the four concurrent group types was designed to consist of 40 participants. Four blocks were run in an attempt to achieve the desired sample size.

Power analyses were conducted using G*Power version 3.1 (48). To test whether stress and anxiety reduced from pre- to post-session, 80% power for detecting a medium effect (*d* = 0.5), at a significance criterion of α = 0.05, was reached at *N* = 41 for a one-tailed paired *t*-test. To test whether state anxiety and stress reductions differed across the therapy and control groups, 80% power for detecting a medium effect (*f* = 0.25), at a significance criterion of α = 0.05, was reached at *N* = 159 for a one-way ANOVA.

To achieve the desired sample of *n* = 160 (40 per block), four blocks were required. To capture student experiences across the school year, the study blocks were run in each of the four semesters. A total of 150 students provided consent to participate in the study, but only 110 students responded to the follow-up emails with questionnaires. The 110 students were randomly assigned to a therapy group or the control group. Students were evenly assigned to the different groups, but as a result of attrition, 84 students (15 males) completed the study: Receptive Music Therapy (*n* = 28), Active Music Therapy (*n* = 18), Verbal Therapy (*n* = 18), Control (*n* = 20). On average, students in the Music Therapy groups attended 77.5% of the online therapy sessions and students in the Verbal Therapy groups attended 71.0% of the online therapy sessions.

Demographically, students from all university Faculties were represented, but most students were in the Faculty of Science (56%). A total of 72/84 students self-described their ethnicity, broadly reporting: 32 Asian, 14 White, 6 African, 6 European, 6 cross-continent, 6 North American, 1 Caribbean, and 1 Jewish (more specific self-descriptions are presented in Supplementary material 2). Ethnicity was not used in the analysis and is presented to characterize the sample.

### 2.3 Procedure

A recruitment poster and recruitment email were circulated via social media platforms and email prior to each 6-week block. Students who responded to the recruitment messages were provided with the consent form as a Google form via email to review. Students choosing to sign and submit the consent form received a link to complete a demographic survey, the Goldsmith Music Sophistication Index (GOLD-MSI), the Ten Item Personality Inventory (TIPI), PSS, and WHO-QOL-BREF (see below for details of these measures). The PSS and WHO-QOL were completed again in week 6 of the study. Prior to the 6-week block commencing, participants received two hair sample collection kits, and were asked to provide a hair sample in week 1 and week 6 of the study. Participants were provided with an ID number to use for data collection to de-identify participants. The kits included instructions and an envelope to mail their hair sample to the lab. Finally, a 6-week recurring zoom link was sent to all participants in the three online therapy groups.

All three online therapy groups were facilitated by a registered psychotherapist, meaning the facilitators were members in good standing with the College of Registered Psychotherapists of Ontario. The therapists facilitating the online music therapy groups were also registered music therapists in good standing with the Canadian Association for Music Therapists. To minimize facilitator effects, different therapists facilitated different blocks throughout the research study, with a total of four music therapists and three verbal therapists participating. In addition, there were three undergraduate student co-researchers per block, who were either completing a research project course for credit or volunteering. Prior to data collection, online practice sessions with student co-researchers and therapists were conducted to review the data collection process.

Each online therapy group session was conducted on Zoom and began and finished with the student co-researcher being present on zoom to help participants as needed to fill out their Google form, which included completing the STAI-S, the Likert stress scale, and measuring their HRV (via the Welltory App on their phone) and recording it. The de-identified data from each participant was automatically input into a spreadsheet for later analysis. During this data collection, participants connected privately if needed with the student co-researcher using the private chat function in Zoom. Each week, after initial data collection, the therapist facilitated a 45 min online group session. The student co-researcher remained in a break-out zoom room during the therapy session and was not present for any of the therapy sessions. At the end of the therapy session, the student co-researcher was again available to help participants fill out their forms.

The instructions for the collection of the hair sample in week 1 and week 6 included the following steps: (1) cutting a small sample of hair and placing it on the paper provided in the kit, (2) folding the paper, and placing the paper with the hair in the pre-addressed, stamped envelope provided, and (3) posting the hair sample to the Drug Safety Laboratory at Western University, Ontario (Supplementary material 3).

Interventions implemented in the online active music therapy group included song writing, singing, lyric analysis, and verbal processing. Interventions implemented in the online receptive music therapy group included participant-directed music listening and verbal processing. Interventions implemented in the online verbal therapy group included verbal processing. Both the online music therapy and verbal therapy groups were informed by the model offered at the McMaster Student Wellness Centre, *Stress Less* (Supplementary material 4).

### 2.4. Measures

Three stress and anxiety measures were collected pre and post each online therapy session for each of the three therapy groups in each block: (1) STAI-S, (2) Likert stress, and (3) HRV. Two stress measures were collected in week 1 and week 6 from the three therapy groups and the control group: (1) PSS and (2) Cortisol. Three standardized questionnaires were given to capture participant variables that could moderate stress and anxiety outcomes: (1) Ten Item Personality Inventory (TIPI), (2) GOLD-MSI, and (3) World Health Organization Quality of Life (WHO-QOL) (49). The first two were collected prior to the onset of the therapy groups. The WHO-QOL was collected in week 1 and week 6 of the study. Participant variables collected from the demographic questionnaire included: self-described gender, self-described ethnicity, year of birth, University Faculty, previous or present use of psychotropic medication, and previous engagement in therapy. The following contains details about each of the measures.

#### 2.4.1. Pre- and post-therapy session measures: all three therapy groups (no control group)

##### 2.4.1.1. State-Trait Anxiety Inventory, State version

The STAI-S includes twenty questions assessing the intensity of participant anxiety at the moment of testing (50). The STAI-S was administered in the present study to measure how students’ anxiety changes as a result of external factors in the moment. When completing the STAI-S, participants rate the intensity of their feelings on a Likert scale from (1) *not at all* to (4) *very much so*. The STAI-S has shown good reliability and validity across different normative groups; Cronbach’s alpha = 0.86–0.95 (50). Construct validity was established in two studies by comparing the mean STAI-S scores of college students in anxiety-inducing conditions (50).

##### 2.4.1.2. Likert stress (1–5)

The Likert scale is an example of a psychometric scale that is flexible and need-based, and whose validity is driven by the applicability of the topic in the context of participant understanding (51). In the present case, participants rated their stress from 1 to 5 (1 = None, 2 = Mild, 3 = Moderate, 4 = High, 5 = Extreme).

##### 2.4.1.3. Heart rate variability

Heart rate variability is a non-invasive measure of the ANS as a reliable assessment of stress (40). Greater variability in heart rate can result in a greater ability to rapidly cope with uncertain and changing environments (41). In this study, HRV was collected using the Welltory smart phone application using the camera of the smart phone. Participants place their finger over the phone camera and flash for 2 min. A previous study compared HRV measurements using the Welltory App and the Polar chest strap (which are ECG accurate-site) and determined the technical error of estimate (TEE) was acceptable for all conditions (average TEE CV% [90% CI] = 6.35 [5.13; 8.5]) and both the PPG- and heart-rate-sensor-derived measures had almost perfect correlations with ECG *(r* = 1.00 [0.99; 1.00]) (52).

#### 2.4.2. Pre-post 6-week intervention measures: all groups

##### 2.4.2.1. Perceived Stress Scale (PSS-10)

The PSS is a 10-item self-report questionnaire designed to evaluate the extent to which an individual perceives life to be “unpredictable, uncontrollable, and overloading” (53). The scale is designed to assess feelings about life events and situations over the previous month using a five-point scale ranging from (0) *Never* to (4) *Very Often*. PSS scores have demonstrated adequate reliability (α = 0.78) and moderate concurrent criterion validity with the amount of stress experienced during an average week (*r* = 0.39, *p* < 0.001) and the frequency of stressful life events within the past year (*r* = 0.32, *p* < 0.001) (54). Additional studies reporting the PSS-10 to have good internal consistency and reliability include Barbosa-Leiker et al. (55), Golden-Kreutz et al. (56), and Reis et al. (57).

##### 2.4.2.2. Cortisol

Cortisol is a glucocorticoid secreted from the adrenal glands that is often used as a biomarker for stress (58). Hair cortisol is not an acute marker of hypothalamic pituitary adrenal axis (HPA-axis) activity. Rather, it acts as a proxy for total HPA activity in the preceding months (45). Cortisol from hair samples thus provides information about participant HPA activity retrospectively. Several studies have shown that hair cortisol levels can serve as a reliable approximation of average blood cortisol levels, pointing to the validity of this method relative to established standards (45, 59).

#### 2.4.3. Standardized questionnaires for participant variables: all groups

##### 2.4.3.1. Ten Item Personality Inventory

The TIPI is a self-report questionnaire consisting of ten pairs of words to measure a person’s Big Five personality dimensions: Extraversion, Agreeableness, Conscientiousness, Emotional Stability, and Openness to experiences (60). Participants are asked to rate the extent that each pair of words applies to themselves on a Likert scale from (1) *disagree strongly* to (7) *agree strongly*. The TIPI has been shown to have good validity: mean convergent validity with the Big-Five Inventory was *r* = 0.77 (60).

##### 2.4.3.2. The Goldsmith Music Sophistication Index

The GOLD-MSI is a psychometric tool for the measurement of musical attitudes, behaviors, and skills. It is comprised of a self-report questionnaire measuring musical sophistication, defined as musical skills, expertise, achievements, and related behaviors (61). There are five subscales within the GOLD-MSI: (1) Active Engagement, (2) Perceptual Abilities, (3) Musical Training, (4) Singing Abilities, and (5) Emotions. A study by Müllensiefen et al. (61), reported that the GOLD-MSI possesses good reliability on each subscale (all α and ω > 0.79).

##### 2.4.3.3. World Health Organization Quality of Life

The WHO-QOL-BREF is a questionnaire containing 26 questions to assess four domains: (1) Physical Health, (2) Psychological Health, (3) Social Relationships, and (4) Environmental Quality of Life. The WHO-QOL-BREF provides a valid and reliable alternative to the assessment of domain profiles using the WHO-QOL-100 (WHO/HIS/HSI Rev.2012.03) (49).

### 2.5. Analysis plan

Analyses were conducted using both JASP 0.14.1 and RStudio 2022.07.02.

#### 2.5.1. Question 1: Does participating in a 45-min online group music therapy session reduce stress (Likert stress) and anxiety (STAI-S) from pre- to post-session in comparison to the corresponding standard of care (online verbal therapy)?

One-tailed paired *t*-tests (corrected for multiple comparisons) were conducted between the average pre-session scores to the average post-session scores for each of the three therapy groups to determine if stress (Likert stress) and anxiety (STAI-S) reduced from pre- to post-session for each of the therapy groups.

Separate one-way ANOVAs were conducted to determine if the average change (pre-session scores were subtracted from post-session scores) in stress (Likert stress) and anxiety (STAI-S) scores differed amongst the three therapy groups (Active Music Therapy, Receptive Music Therapy, and Verbal Therapy). Following this, Bayesian ANOVAs were conducted to determine the degree of evidence for the null hypothesis (i.e., no difference among the three therapy groups in stress and anxiety reduction).

#### 2.5.2. Question 2: Does participating in 6 weeks of weekly online group music therapy session reduce stress in comparison to the corresponding standard of care (online verbal therapy) and to the no-intervention control group?

Separate one-tailed paired *t*-tests (corrected for multiple comparisons) were conducted to determine whether there was a reduction in PSS and in cortisol scores between week 1 and week 6 scores for each of the therapy groups and the control group.

Separate one-way ANOVAs were planned to determine if the difference in PSS and cortisol scores across the 6 weeks (week 1 scores were subtracted from week 6 scores for each group) differed among the two music therapy groups and the control group. Following this, Bayesian ANOVAs were conducted to determine the degree of evidence for the *a priori* null hypothesis that there was no difference among the two music therapy groups in comparison to the standard of care (verbal therapy group) in stress reduction based on PSS and cortisol scores.

## 3. Results

### 3.1. Outcome variables: stress and anxiety

#### 3.1.1. Question 1: Does participating in a 45-min online group music therapy session reduce stress (Likert stress) and anxiety (STAI-S) from pre- to post-session in comparison to the corresponding standard of care (online verbal therapy)?

A total of 64 students participated in the therapy groups and provided STAI-S and Likert stress scores pre and post each group therapy session. The pre- vs. post-session scores for STAI-S and Likert stress met assumptions for equal variance [*F*(2,61) = 0.85, *p* = 0.43; *F*(2,61) = 1.47, *p* = 0.24, respectively].

One-tailed paired *t*-tests comparing the average pre- and the average post-session STAI-S and Likert stress scores for each online group therapy session revealed a significant average reduction in both STAI-S and Likert stress scores separately for each of the three therapy groups (all *p*’s < 0.0008 after Bonferroni Correction for multiple comparisons). Detailed results are presented in Table 1.

**TABLE 1:** Change in stress and anxiety scores pre to post sessions (for each participant, their pre-score minus post-score was averaged across sessions).

		STAI-S						Likert stress					
Group*	***N* (males)**	**Pre** **(SD)**	**Post** **(SD)**	**Change (SD)**	***t***	**df**	***d***	**Pre** **(SD)**	**Post (SD)**	**Change (SD)**	***t***	**df**	***d***
AMT	18 (2)	43.20 (8.94)	32.92 (6.90)	−10.28 (5.28)	7.4	17	1.77	3.03 (0.81)	2.26 (0.64)	−0.77 (0.63)	5.18	17	1.22
RMT	28 (5)	44.59 (9.09)	34.89 (6.72)	−9.70 (5.5)	9.3	27	1.77	3.09 (0.93)	2.23 (0.81)	−0.86 (0.69)	6.55	27	1.24
VT	18 (3)	45.29 (10.85)	37.08 (8.07)	−8.21 (7.48)	4.6	17	1.10	3.29 (0.68)	2.63 (0.68)	−0.65 (0.64)	4.43	17	1.04
All	64 (10)	44.40 (9.46)	34.95 (7.23)	−9.45 (6.15)	12.3	63	0.54	3.15 (1.43)	2.37 (0.94)	−0.78 (0.66)	8.58	63	1.07

All *p*’s < 0.001; Bonferroni alpha = 0.0083 (0.05/6 comparisons). *AMT, Active music therapy; RMT, receptive music therapy; VT, verbal therapy.

ANOVAs using the average change scores (pre-session scores subtracted from post-session scores) for each therapy group found no significant differences among therapy groups for either change in STAI-S scores [*F*(2,61) = 0.55, *p* = 0.58, η^2^ = 0.02] or Likert stress scores [*F*(2,61) = 0.09, *p* = 0.91, η^2^ = 0.003]. To provide stronger evidence for the null hypothesis (no difference between the music therapy groups and the verbal therapy standard of care), a Bayesian ANOVA revealed moderate evidence that the therapy groups did not differ from the standard of care on either changes in STAI-S (BF_10_ = 0.195) or Likert stress (BF_10_ = 0.198) scores.

Due to the HRV scores being highly variable, only descriptive statistics are reported (see Supplementary material 4). According to the app used, HRV should range from 65 to 105 ms (62). Our participants reported measurements from 3.3 to 298 ms. This variability was likely due to several factors, including a lack of control of participants’ activities at home immediately preceding the measurements, perhaps not using the app correctly, and potential issues in participants’ reporting of the HRV values from the Welltory phone application.

#### 3.1.2. Question 2: Does participating in 6 weeks of weekly online group music therapy sessions reduce stress in comparison to the corresponding standard of care (online verbal therapy) and to a no-intervention control group?

A total of 68 students completed the PSS in both week 1 and week 6, and a total of 39 students provided hair samples in both week 1 and week 6. The cortisol data were heavily skewed; therefore, we log-transformed the cortisol data (63, 64). One-tailed paired *t*-tests comparing week 1 scores to week 6 scores for PSS revealed only a significant average reduction in PSS scores in the Receptive Music Therapy group (*p* = 0.02), but the finding did not survive Benjamini–Hochberg correction for multiple comparisons (*p* = 0.08). One-tailed paired *t*-tests comparing week 1 cortisol to week 6 cortisol for each group revealed only a significant increase in cortisol in the control group (*p* = 0.04 after Benjamini–Hochberg corrections for multiple comparisons). Detailed results are presented in Table 2.

**TABLE 2:** Change in perceived stress and cortisol week 1 to week 6.

	PSS						Cortisol						
Group*	***N* (males)**	**Change (SD)**	***p***	***t***	***df***	**Effect**	***N* (males)**	**Change (SD)**	***p***	***t* (W)**	***df***	**Effect**	**Shapiro–Wilk**
AMT	16 (1)	−1.94 (6.63)	0.13	1.2	15	0.29	8 (1)	−0.05 (0.25)	0.31	0.54	7	0.19	0.53
RMT	19 (3)	−2.90 (5.48)	0.02	2.3	18	0.53	11 (2)	−0.11 (0.23)	0.06	1.67	10	0.5	0.40
VT	15 (3)	−1.80 (5.81)	0.13	1.2	14	0.31	9 (1)	−0.00 (0.71)	0.33	(27)	8	0.2	0.016**
C	18 (4)	−0.78 (4.67)	0.76	0.71	17	0.17	11 (3)	0.31 (0.49)	0.01	(8)	10	−0.76	0.005**

For the Student’s t-test, effect size (Cohen’s *d*). For the Wilcoxon test, effect size (matched rank biserial correlation). *AMT, Active music therapy; RMT, receptive music therapy; VT, verbal therapy. **Wilcoxon signed-rank used for Shapiro–Wilk *p* < 0.005.

To test our hypothesis that reductions in PSS scores would differ among the music therapy groups (active music therapy group, receptive music therapy) and the control group, we conducted a one-way ANOVA comparing difference scores (week 1 scores were subtracted from week 6 scores). We found a non-significant effect of group [*F*(2,50) = 0.661, *p* = 0.521, η^2^ = 0.026], indicating that we found no evidence that the music therapy groups differed significantly from the control group for changes in PSS scores from week 1 to week 6.

To determine whether the two music therapy groups (active music therapy group, receptive music therapy) were equivalent on PSS change scores to the standard of care (verbal therapy), a Bayesian ANOVA revealed moderate evidence that the two music therapy and the verbal groups did not differ (BF_10_ = 0.126).

With respect to our hypothesis that reductions in cortisol scores would differ among the music therapy groups (active music therapy group, receptive music therapy) and the control group, difference scores (week 1 scores were subtracted from week 6 scores) failed to meet the Shapiro-Wilk criteria for normality (W_*Pre*_ = 0.89, *p* < 0.01; W_*Post*_ = 0.91, *p* < 0.01; W_*Cortisoldifference*_ = 0.83, *p* < 0.01). Therefore, the non-parametric Kruskal–Wallis test was used to compare the cortisol difference scores from among the two music therapy groups and the control group. This revealed a significant effect of group (receptive music therapy, active music therapy, and control group) on change in cortisol [Kruskal–Wallis χ^2^(2) = 7.73, *p* = 0.02, η^2^ = 0.25]. A pairwise *post-hoc* Dunn test with Bonferroni adjustments revealed significant differences between the receptive music therapy group and the control group (*p* = 0.01).

To determine whether the two music therapy groups (active music therapy group, receptive music therapy) and the standard of care (verbal therapy group) were equivalent on cortisol difference scores, a Bayesian ANOVA revealed anecdotal evidence that therapy group had no effect on the changes in cortisol (BF_10_ = 0.655).

### 3.2. Correlates of stress and anxiety outcomes

Pearson correlations across all possible participants (i.e., collapsing across the three therapy groups for the pre-post session scores, and all four groups for the pre-post intervention period scores) were conducted between each participant variable and the four stress and anxiety difference scores (Table 3). After corrections for multiple comparisons, the only significant correlation was between changes in PSS and the personality trait of conscientiousness (*r* = 0.39, *p* = 0.02). The direction of the relationships was such that higher conscientiousness was related to an average increase in PSS scores across the 6-week intervention period.

**TABLE 3:** Correlates of stress and anxiety difference scores.

	Participant variables	*r*	*p* *
**Stress anxiety change**			
State anxiety	Block of study	0.11	0.40
Likert	Block of study	0.29	0.21
PSS	Block of study	-0.10	0.37
Cortisol	Block of study	-0.10	0.55
State anxiety	Gender	0.11	0.38
Likert	Gender	-0.18	0.16
PSS	Gender	-0.15	0.23
Cortisol	Gender	-0.03	0.84
State anxiety	Attendance	-0.16	0.22
Likert	Attendance	-0.37	0.08
PSS	Attendance	-0.07	0.63
Cortisol	Attendance	-0.45	0.21
State anxiety	Year of birth	0.07	0.58
Likert	Year of birth	0.17	0.21
PSS	Year of birth	-0.26	0.21
Cortisol	Year of birth	0.18	0.30
State anxiety	University faculty	-0.12	0.36
Likert	University faculty	-0.27	0.21
PSS	University faculty	0.17	0.18
Cortisol	University faculty	-0.19	0.25
State anxiety	Medication	0.05	0.70
Likert	Medication	0.22	0.09
PSS	Medication	0.04	0.73
Cortisol	Medication	0.03	0.87
State anxiety	Therapy	-0.09	0.52
Likert	Therapy	0.22	0.09
PSS	Therapy	0.19	0.13
Cortisol	Therapy	-0.12	0.48
**TIPI**			
State anxiety	Extroversion	-0.06	0.67
Likert	Extroversion	-0.13	0.35
PSS	Extroversion	-0.04	0.74
Cortisol	Extroversion	0.18	0.30
State anxiety	Agreeableness	0.04	0.76
Likert	Agreeableness	-0.03	0.84
PSS	Agreeableness	0.16	0.20
Cortisol	Agreeableness	-0.03	0.87
State anxiety	Conscientiousness	-0.03	0.84
Likert	Conscientiousness	-0.02	0.91
PSS	Conscientiousness	0.39	0.02*
Cortisol	Conscientiousness	0.13	0.44
State anxiety	Emotional stability	0.00	0.99
Likert	Emotional stability	-0.16	0.23
PSS	Emotional stability	0.04	0.78
Cortisol	Emotional stability	-1.70	0.32
State anxiety	Openness	-0.28	0.29
Likert	Openness	-0.27	0.29
PSS	Openness	-0.03	0.82
Cortisol	Openness	0.10	0.60
**QOL**			
State anxiety	Physical	-0.17	0.27
Likert	Physical	-0.01	0.38
PSS	Physical	-0.07	0.62
Cortisol	Physical	0.06	0.73
State anxiety	Psychological	-0.17	0.30
Likert	Psychological	-0.01	0.94
PSS	Psychological	-0.36	0.06
Cortisol	Psychological	0.04	0.82
State anxiety	Social	-0.34	0.21
Likert	Social	-0.27	0.08
PSS	Social	-0.17	0.18
Cortisol	Social	0.16	0.35
State anxiety	Environmental	-0.14	0.37
Likert	Environmental	-0.13	0.41
PSS	Environmental	-0.01	0.92
Cortisol	Environmental	-0.15	0.39
**GOLD-MSI**			
State anxiety	Active engagement	0.11	0.45
Likert	Active engagement	-0.08	0.57
PSS	Active engagement	-0.25	0.06
Cortisol	Active engagement	0.24	0.15
State anxiety	Perceptual abilities	0.04	0.80
Likert	Perceptual abilities	-0.04	0.78
PSS	Perceptual abilities	-0.25	0.06
Cortisol	Perceptual abilities	-0.08	0.67
State anxiety	Musical training	-0.11	0.43
Likert	Musical training	-0.25	0.07
PSS	Musical training	-0.02	0.85
Cortisol	Musical training	0.09	0.60
State anxiety	Singing abilities	0.05	0.73
Likert	Singing abilities	-0.12	0.38
PSS	Singing abilities	-0.07	0.58
Cortisol	Singing abilities	0.07	0.67
State anxiety	Emotions	0.11	0.42
Likert	Emotions	0.15	0.29
PSS	Emotions	-0.16	0.22
Cortisol	Emotions	0.03	0.90
State anxiety	General music sophistication	0.01	0.93
Likert	General music sophistication	-0.15	0.29
PSS	General music sophistication	-0.12	0.36
Cortisol	General music sophistication	0.10	0.54

The variable *Medication* refers to the participants’ past or present use of psychotropic medication, and the variable *Therapy* refers to past or present experience with therapy.

*p < 0.05 with Benjamini–Hochberg corrections for multiple comparisons.

## 4. Discussion

In alignment with the World Health Organization’s (WHO) goal to provide comprehensive and integrated mental health services in community-based settings, this research explored the efficacy of online group music therapy as a proactive intervention for reducing university students’ stress and anxiety. The term proactive refers to engaging students in therapy as a means to manage the stressors and anxiety of student life. Stress can be a healthy emotion when an individual has the tools to manage it, whereas persistent exposure to stressors and continual activation of the stress response can be detrimental to health and wellbeing (65). To our knowledge, this RCT reports on the first application of online group music therapy for proactive wellness with undergraduate university students.

Regarding our first question, *whether participating in a 45-min online group music therapy session reduces stress and anxiety in comparison to the corresponding standard of care (online verbal therapy)*, we found that both STAI-S and Likert stress self-report scores decreased significantly from pre to post therapy session, for each therapy group (active music therapy, receptive music therapy, and verbal therapy). Furthermore, there were no significant differences among the groups and a Bayesian analysis found moderate evidence for no difference among the groups. Thus, by these self-report measures, group music therapy was effective and no different from the standard of care (online verbal therapy). As far as a direct measure of ANS function, this was more challenging to collect remotely. Unfortunately, the HRV scores reported by participants using a phone app at home appeared to be unreliable, so it is difficult to make any conclusions regarding this measure. However, the evidence from the STAI-S and Likert stress tools clearly points to short-term benefits of online group music therapy that are similar to the verbal therapy standard of care.

Regarding our second question, *whether participating in 6 weeks of weekly online group music therapy sessions reduces stress in comparison to the corresponding standard of care (online verbal therapy) and a no-intervention control group*, there were no significant differences among the four groups (active music therapy, passive music therapy, verbal therapy, and control) on the self-report PSS stress scale, and a Bayesian analysis found moderate evidence that the groups did not differ. Furthermore, changes in PSS from week 1 to week 6 were not significantly different from chance for any group when corrected for multiple tests. The research comparing self-reported stress scores to a biomarker is mixed; several studies have reported non-significant changes in self-reported measures of stress, despite reporting significant changes in cortisol (44, 66), although several also report significant correlations between self-reported stress and cortisol (67, 68). Regarding cortisol, despite only about half of participants sending in both hair samples, there was a significant difference among the music therapy groups and the control group on the change in cortisol across the intervention. *Post-hoc* tests revealed that the receptive music therapy and control groups differed significantly on cortisol changes. Specifically, this was driven by a significant increase in cortisol in the control group and a marginal decrease in cortisol in the receptive music therapy group, as revealed via paired *t*-tests. Without intervention, it is plausible that stress levels would increase over the university term, as they did in the control group. In this light, it is interesting that the intervention groups did not show this trend. Future studies should attempt to replicate these findings with a larger sample size.

Individual differences have been reported as an important factor in the experience of stress (2). We therefore examined several variables that might moderate stress and anxiety outcomes. None of the demographic variables collected, including gender, and area of study at university, correlated significantly with any of the measures of change in stress or anxiety after correcting for multiple comparisons. Although there is literature relating personality traits to stress responses, the role of personality in response to engaging in therapy has been less studied. A meta-analysis on the associations between the Big Five personality traits and stress reported neuroticism was positively related to stress, while extraversion, agreeableness, conscientiousness, and openness were negatively related to stress (69). In the present research study, only conscientiousness was significantly correlated with changes in PSS after correction for multiple comparisons. We found that conscientiousness scores were significantly correlated with an increase in PSS scores from week 1 to week 6, suggesting that people with this personality trait may be less responsive to therapy. However, the present study differed from previous studies that explored correlations between PSS scores and personality as it explored *changes* in PSS scores over the study period, as opposed to PSS scores in the final week of the study, which appears to be a more common study design. Given that these analyses were exploratory, further research is needed to understand the complex relations between various individual differences and responsiveness to music therapy.

Participants also completed the QOL questionnaire as stress and anxiety levels can be affected by particular events in an individual’s life that affect their quality of life. We found no significant associations between any of the QOL subscales and any of the measures of stress and anxiety after correcting for multiple comparisons. However, as the study was conducted during the COVID-19 pandemic restrictions, it is possible that all or most students were experiencing negative quality of life changes, making it difficult to see effects of individual differences. Given that previous studies have linked lower quality of life scores to higher stress (70–72), it would be useful for future research to examine how quality of life measures might relate to music therapy outcomes with a larger sample size and outside of a pandemic period.

Engaging in music therapy does not require participants to have a background in music, or to be able to play an instrument or to sing. However, whether musical sophistication affects music therapy outcomes remains understudied. We did not observe any significant correlations between music sophistication scores and any of the stress or anxiety measures, nor did we find any significant differences between the active and receptive music interventions. While these null findings need to be replicated with a larger sample, they suggest that musical sophistication may not be necessary for positive music therapy outcomes and that participants with varied musical backgrounds may benefit even from music therapy that involves active music making.

Despite several challenges and limitations (see section “4.1. Limitations”), the present study was innovative in showing that music therapy can be effectively delivered to university students online and in a group setting. The COVID-19 pandemic has likely forever changed aspects of health care delivery. Beyond the scope of university students, access to proactive online group mental health therapies provides a relatively inexpensive option that can drastically increase accessibility for many populations, including those from poorer economic backgrounds, those who have mobility challenges (such as seniors in care), and those living in remote areas (32).

Although on-campus treatment options are being expanded in Canada, few universities have attempted a whole campus approach to create a health-promoting culture, as is described in the Okanagan Charter (73), and we are not aware of any campuses that are offering music therapy. One important aspect of a health-promoting cultures is a *proactive approach* that provides services aimed at improving mental health before crisis situations are reached. This is of course beneficial for students, but at the same time it could lead to reductions in treatment costs. A second important aspect of a health-promoting culture is to *include a variety of options*. Creative arts therapies are not recognized as a standard of care for mental health goals, yet the findings of the present research suggest that music therapy could be a viable option to offer to students on a university campus. Considering that 75% of students who experience significant psychological distress do not seek support as a result of negative stigma (18), offering music therapy could help to lower this number as a non-negatively stigmatized option for support. This idea is further supported by a survey completed by 786 university students who indicated the most interest in engaging in music therapy for mental health support, followed by art therapy, and lastly verbal therapy (11). In sum, the present results support that the option of online group music therapy on campus for students without a clinical diagnosis can effectively reach some students who would not otherwise engage in proactive therapy for stress and anxiety.

### 4.1. Limitations

Conducting this online study presented some challenges. First, because the university campus was closed as a result of COVID-19 restrictions, students had to be recruited remotely, and we experienced a considerable attrition rate from the time students completed the consent form and pre-questionnaires, to the time the time the therapy sessions began. Thus, our sample size was smaller than desired, affecting statistical power, particularly for between-group analyses. Collecting the important physiological data was also a challenge remotely. Although participants were instructed on how to collect HRV data using an app on their phone, we were not able to control how well they did this, the accuracy of their reporting, or what activity they were engaged in immediately prior to the therapy session. In the end, the HRV data was highly variable and not analyzable. Future online studies will need to find a more reliable method to collect this data. Finally, although the cortisol analyses yielded significant results, only about half of the participants sent in both samples, so these analyses were underpowered.

## 5. Conclusion

The present randomized control trial conducted during COVID-19 restrictions highlights the benefits of offering online group music therapy to university students as a proactive intervention for stress and anxiety in the absence of a clinical diagnosis. Significant reductions in anxiety, as measured by the STAI-S, and stress, as measured on a Likert scale, were observed from pre- to post-45 min of both active and receptive online group music therapy. Further, there was moderate evidence that these reductions in stress and anxiety did not differ from the standard of care (online verbal therapy), suggesting that group music therapy provides a viable option for stress and anxiety reduction. Significant reductions in stress from week 1 to week 6 were not observed by the PSS report measure. However, cortisol levels measured from hair samples taken at the beginning and at the end of the therapy period significantly increased in the control group from week 1 to week 6 as the university term progressed but remained stable in the therapy groups from week 1 to week 6. This study is unique in targeting university students without a clinical diagnosis and exploring the efficacy of online music therapy relative to the standard of care. Further, it is the first music therapy study to measure cortisol from hair samples collected remotely, pushing the boundaries of remote physiological measurement in therapy assessment. The results suggest a choice in therapy type could benefit many students and that music therapy can provide an alternative for students reluctant to engage in, or unable to access, verbal therapy options. This study provides an example of how a health-promoting culture on university campuses can be achieved, consistent with the mental health goals of the Okanagan Charter.

## Data availability statement

The raw data supporting the conclusions of this article will be made available by the authors, without undue reservation.

## Ethics statement

The studies involving human participants were reviewed and approved by the Hamilton Integrated Research Ethics Board (HiREB#11376). The patients/participants provided their written informed consent to participate in this study.

## Author contributions

RF and LT designed the study. RF supervised the implementation of the protocol and data collection. RF and SM analyzed the data with input from LT. All authors contributed to writing the manuscript, read, and approved the submitted version.
