# Supplementary material for: Online group music therapy: proactive management of undergraduate students’ stress and anxiety
Source: Front Psychiatry. 2023 Apr 21;14:1183311. doi: 10.3389/fpsyt.2023.1183311 (PMC10160410; doi:10.3389/fpsyt.2023.1183311)
Supplement: Supplementary file 1 [file Data_Sheet_1.PDF]

frontiers

Supplementary Material

Online Group Music Therapy:
Proactive Management of Undergraduate Students' Stress and Anxiety

Finnerty, R, McWeeny, S, and Trainor, L

1. <u>On-Line Group Therapy Guidelines</u>

Adopted with permission from McMaster University Open Circle

- **Give and receive welcome.** People learn best in hospitable spaces. In this space we support each other's learning by giving and receiving hospitality.
- **Respect**: Listen to each other with openness and curiosity, being respectful of different ideas and opinions while open to learn from everyone.
- **No fixing, saving, advising or correcting each other.** This is one of the hardest guidelines for those of us who like to "help." But it is vital to welcoming the soul.
- **Set aside reaction and judgment and turn towards wonder and compassionate inquiry**. Ask yourself, "I wonder why they feel/think this way?" or "I wonder what my reaction teaches me about myself?" Set aside judgment to listen to others—and to yourself—more deeply.
- **Speak your truth in ways that respect each person's truth.** Our views of reality may differ but speaking one's truth in this space does not mean interpreting, correcting or debating what others say. Speak from your center to the center of the group, using "I" statements, trusting people to do their own sifting and growth.
- **Creating inclusive space for diversity.** We work together to hold a safe space for all forms of diversity, including ethnicity, religion, gender identity and expression, sexual orientation, ability, and socio-economic status.
- **Freedom to share or to pass.** Be here with your listening as well as your speaking.
- **Be present as fully as possible.** Turn off phones and put away technology that is not being used for participation in the group.
- **Trust and learn from the silence.** Silence is a gift in our noisy world, and a way of knowing in itself. Treat silence as a member of the group. After someone has spoken, take time to reflect without immediately filling the space with words.
- **Observe deep confidentiality.** Safety is built when we can trust that our words and stories will remain with the people with whom we choose to share and are not repeated to others without our permission. Do not record any of the groups.

2. Self-described Ethnicity

Self-Described Ethnicity	Broad Description
South Asian	African
Philippines	Asian
African	Asian
South Asian	Asian
East Asian	Asian
South Asian	Asian
Vietnamese	Asian
East Asian	Asian
South Asian	Asian
Chinese	Asian
Pakistan	Asian
I am of Afro-Caribbean descent	Caribbean
Ashkenazi Jewish	Jewish
White/Hispanic	Cross-Continents
Canadian/ American	North American
Caucasian	White
White	White
Caucasian	White
Black/Caribbean/African	African
Chinese	Asian
Chinese	Asian
Chinese	Asian
indian	Asian
Arab	Asian
My Father (100% Greek) immigrated for Greece as a child and met my mother (unknown) in Canada I identify and relate heavily to Greece culture	European
Polish	European
Honduras/USA	Cross-Continents
Moroccan, Canadian	Cross-Continents
Canadian	North American
Canadian	North American
Caucasian	White
Caucasian	White
White	White

White (Caucasian)	White
White	White
Egyptian	African
African	African
African	African
Pujabi-Indian	Asian
Chinese	Asian
Asian	Asian
Family from Pakistan, I was born and raised in Canada	Asian
Chinese and Japanese	Asian
Asian	Asian
South Asian(Indian)	Asian
Chinese	Asian
Caucasian, European	European
European	European
European/Dutch/Scottish	European
Irish/Metis	Cross-Continents
Indian/African	Cross-Continents
Middle Eastern,European	Cross-Continents
Chinese	North American
Canadian	North American
Caucasian	White
White	White
White Caucasian	White
Caucasian	White
Egyptian/Coptic/North African,	African
Chinese	Asian
Afghanistan	Asian
Chinese	Asian
South Asian	Asian
Iranian	Asian
Chinese	Asian
Chinese	Asian
Persian	Asian
Taiwanese	Asian
European/Caucasian	European
English Canadian, Italian	North American
White/Caucasian	White
Caucasian	White

3. Hair Collection Instructions

STEP 1: GATHER METERIALS FOR SAMPLE COLLECTION.

You will need: Sharp and clean scissors, Clear scotch tape, Hair clip (optional), Pen

STEP 2: LOCATE THE POSTERIOR VERTEX REGION OF THE SCALP.

This region of the head has the most consistent hair growth rate. Sampling from here will minimize the variation in sample measurements.

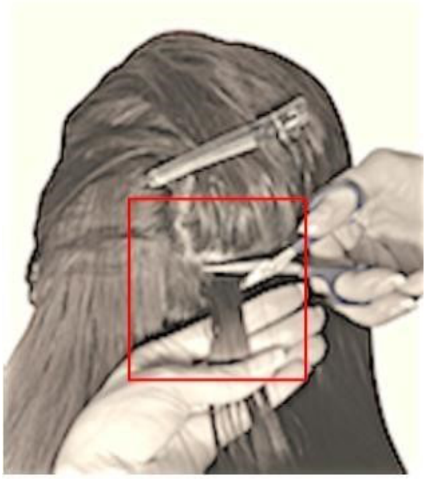

STEP 3: ISOLATE HAIR WITH HAIR CLIP (OR FINGERS) & CUT HORIZONTALLY WITH SCISSORS AS CLOSE TO SCLAP AS POSSIBLE.

Approx. 100 strands of hair are required, 1cm in length, 5mm in diameter (thickness of pencil eraser). Please ensure hair strands are aligned and secured with tape to the direction of the scalp and that the direction of the scalp is clearly indicated. Please use the collection form provided.

STEP 4:TAPE THE SCALP END OF THE HAIR TO THE HAIR SAMPLE COLLECTION FORM & FILL OUT STUDY ID, SUBJECT ID, & COLLECTION DATE.

Clear scotch tape works best. It is easily removed and leaves no residue on the hair. Fold the paper along the length of the hair & place in pre-addressed envelope to secure the sample. Post the pre-addressed envelope after collecting. The pre-addressed envelope will be addressed to Drug Safety Laboratory, Robarts Research Institute, Western University, 1151 Richmond St N, London, On. Canada.

3. *Stress Less* Online Group Description

In keeping with the McMaster Student Wellness group "Stress Less", each group explores the similarity and differences between stress, anxiety, and fear, identify stressors, explore stress physiology, discuss existing stress management strategies such as mindfulness, and introduce progressive muscle relaxation. Verbal facilitation is implemented in all three groups.

*Group guidelines reviewed	**Opening Quote & Ice Breaker**	**Facilitated reflection on a theme related to stress and anxiety**	**Closing Activity & Quote**
Verbal Based Therapy Group	Verbal Check-in.	Journaling, followed by group sharing.	Verbally facilitated progressive muscle relaxation.
Music Therapy (Active)	Therapist will sing and self-accompany with an instrument.	Lyric creation facilitated for a piece of music using either a pre-composed or original melody. Participants will have the option to sing along.	Live music facilitated progressive muscle relaxation.
Music Therapy (Receptive)	Pre-recorded music will be played.	Using music listening (choice of music guided by participants)	Music listening facilitated progressive muscle relaxation.

4. Supplemental Tables

Supplementary Table 1
Descriptive statistics for Heart Rate Variability Scores Pre and Post each Therapy Session

	Pre 1	Post 1	Pre 2	Post 2	Pre 3	Post 3	Pre 4	Post 4	Pre 5	Post 5	Pre 6	Post 6
M	67.485	60.913	60.520	65.000	58.813	64.392	65.122	68.683	59.667	70.637	60.565	77.478
SD	43.690	24.005	26.063	25.678	29.974	28.828	25.619	24.810	28.121	24.986	26.995	48.660
Minimum	3.300	14.000	14.000	15.000	13.000	4.000	23.000	19.000	10.000	25.000	19.000	27.000
Maximum	298.000	100.000	106.000	106.000	179.000	109.000	105.000	100.000	100.000	115.000	109.000	267.000
